# Authors' Response to Peer Reviews of “Early Experience With Neutralizing Monoclonal Antibody Therapy for COVID-19: Retrospective Cohort Survival Analysis and Descriptive Study”

**DOI:** 10.2196/33496

**Published:** 2021-09-27

**Authors:** Mark Jarrett, Warren Licht, Kevin Bock, Zenobia Brown, Jamie Hirsch, Kevin Coppa, Rajdeep Brar, Stephen Bello, Ira Nash

**Affiliations:** 1 Donald and Barbara Zucker School of Medicine at Hofstra/Northwell Hofstra University Hempstead, NY United States; 2 Northwell Health New Hyde Park, NY United States

**Keywords:** infectious disease, monoclonal antibody therapy, COVID-19, experience, therapy, drug, patient outcome, risk, efficacy, approach, treatment, pandemic, antibody, immunotherapy, immune therapy

*This is the authors’ response to peer-review reports for “Early Experience With Neutralizing Monoclonal Antibody Therapy for COVID-19: Retrospective Cohort Survival Analysis and Descriptive Study.”*

## Round 1 Review [1]

1. Formatting in paper [2] changed as requested
2. Figures changed
3. Added suggested contextualization
4. Fortified Discussion
5. Although we added references and discussion of the inflammatory response to cytokines, it should be recognized that these antibodies work by neutralization of the virus, not by affecting cytokines.
